# NUMERACY SKILL USE AMONG MIDDLE-AGED AND OLDER WORKERS IN THE US

**DOI:** 10.1093/geroni/igac059.2496

**Published:** 2022-12-20

**Authors:** Takashi Yamashita, Donnette Narine, Abigail Helsinger, Wonmai Punksungka, Phyllis Cummins, Jenna Kramer, Rita Karam, Thomas Smith

**Affiliations:** University of Maryland, Baltimore County, Baltimore, Maryland, United States; University of Maryland, Baltimore, Baltimore, Maryland, United States; Miami University, Oxford, Ohio, United States; University of Maryland - Baltimore County, Baltimore, Maryland, United States; Miami University, Oxford, Ohio, United States; RAND Corporation, Arlington, Virginia, United States; RAND Corporation, Santa Monica, California, United States; Northern Illinois University, DeKalb, Illinois, United States

## Abstract

In numeric information-rich societies, numeracy is essential both at work and in everyday life (e.g., calculating a budget). Numeracy skills generally decline with aging. Thus, among middle-aged and older workers, maintaining and improving numeracy skills is crucial to securing employment as well as managing everyday life. One of the counteracting strategies is to practice numeracy. However, little is known about how and what kinds of numeracy skills are used among older workers. We analyzed a nationally representative sample of U.S. workers aged 45 to 74 (n = 3,850) from the 2012/2014/2017 Program for International Assessment of Adult Competencies (PIAAC) restricted-use-file. Six dichotomous numeracy use indicators (e.g., calculating a budget, using advanced statistics) at work and in everyday life were considered. Survey weighted latent class analysis (LCA) identified three subgroups with distinctive numeracy skill use patterns, including ubiquitous (both at work and in everyday life) users, occupational users, and non-users. For example, calculating a budget was common both in non-users (40%) and ubiquitous users (84%), whereas uncommon among occupational users (25%). Also, only ubiquitous users practiced advanced math and statistics (14%), while others did not (nearly 0%). The subsequent regression analysis revealed that higher educational attainment, higher income, certain racial group (i.e., Whites), and better self-rated health were associated with greater numeracy skill use. The lack of numeracy skill use leads to lower skill levels and, in turn, social and economic disadvantages in later life. In addition to more detailed LCA results interpretations, possible policy and educational interventions are evaluated.

